# Progress in Surgery

**Published:** 1898-09-10

**Authors:** 


### Progress in Surgery.

### SURGERY OF NERVES.

**Neuro-fibromata of the Cervical Sympathetic.**—The author of this paper[1] records in detail what he considers a unique case of large multiple neuro-fibromata of the cervical sympathetic. The patient was a girl of nine, who came asking the removal of a large painless growth which extended from the sternal end of the right clavicle up to the level of the hyoid bone. It lay beneath the sterno-mastoid muscle and the large vessels of the neck, was exceedingly hard, and had only been observed for two months. The provisional diagnosis was an unusual tumour of branchial origin.

On exposing the tumour by a longitudinal incision it was found to be in the situation already indicated, of cartilaginous, almost bony consistence, and to have branching, tortuous processes passing in different directions from the main mass. One of these terminated in a bulbous mass. Anatomically it corresponded to nothing except the cervical sympathetic. The whole growth with its processes was dissected out with little trouble, and the wound healed by first intention. The ocular conjunctiva of the right eye was suffused for about a week, and the pupil on that side became immediately and permanently narrowed. There was also a narrowing of the palpebral fissure.

Pathologically the growth was found to be a fibro-sarcoma (*sic*) of nerve trunks, originating in the endoneurium of the nerve sheath.

Although such tumours are common enough in the spinal nerves, they have only hitherto been described from post-mortem cases in the sympathetic. Some cases, however, have been met with clinically secondary to similar growths in the spinal nerves. These are quoted in the paper before us.

**Course and Treatment of Injuries to the Peripheral Nerves.**—At the meeting of the American Medical Association A. H. Levings[2] read a paper on certain experiments he has made on animals to determine the results of nerve section, and the various methods of repairing injuries to the peripheral nerves. All the experiments, 85 in number, were made upon the sciatic nerve, and the following conditions to success in operating are formulated: (1) Asepsis; (2) care in avoiding separation of the nerve from the connective tissue about it; (3) square and even approximation of the ends; (4) the use of tissue with longitudinal fibres to bridge any gap between the ends. Any tissue may be used for this purpose, nerve, silk, catgut, &c., but the best results have been obtained with muscle, and silk is superior to catgut. Success is almost invariable, provided not more than three or four inches of the nerve have been removed.

A clinical case was reported by Powers, in which he used four inches of the sciatic nerve of a dog to bridge over a gap in the musculo-spiral nerve. Sensation returned in three or four weeks, but motion did not.

Reed has been successful, both in animals and man, in restoring function by attaching the divided nerve to a neighbouring one. Levings thinks this proceeding more successful in re-establishing sensory than motor function.

**Removal of the Inferior Dental Nerve through the Mouth.**—In a case of trifacial neuralgia, A. H. Ferguson[3] successfully performed this operation. After dividing the nerve at the mental foramen, a half-inch trephine was applied to the lower jaw opposite the two last molar teeth, and the nerve exposed as it runs through the bone. The nerve was again divided where it enters the bone on the inner aspect of the ascending ramus, and the whole of its intra-osseous portion removed by pulling upon it from the trephine opening. Relief was instantaneous and permanent.

[1] Annals of Surgery, April, 1898. [2] Epit. Brit. Med. Jour., July, 1898. [3] New York Med. Jour., May 28, 1898.

### GENERAL SURGERY.

**Antiseptics.**—Iodoformogen powder—a chemical compound of iodoform and albumin, with very little odour—has lately been employed by E. Kromayer[1] and others. It presents the same favourable properties as iodoform, but also some less favourable, such as irritation of the skin. Its action is more lasting, on account of its iodoform being gradually disengaged. Recent investigations upon chinosol, which was first alluded to in The Hospital of April 18th, 1896, and since been extensively used as a powerful antiseptic, show that it is not so innocuous as at first supposed. The *Lancet*[2] draws attention to Professor Hobday's experiments on dogs, cats, and donkeys. When administered internally the drug possesses toxic properties. If used subcutaneously chinosol, when concentrated, produces local irritation and swelling. The cat is more susceptible to its action than the dog. M. B. Werner[3] gives the following antiseptic treatment of burns, for which he claims excellent results. Place the burned limb in a carbolic bath—2 to 5 per cent., according to the age of the patient and the extent of the injured surface. Antisepsis, asepsis, and anæsthesia are thereby effected. Remove all the acid solution by a second bath in physiological saline solution. Then dust the entire surface with a powder containing acetanilid (antifebrin) (1 part), compound zinc stearate (5 parts), and cover the surface with narrow strips of green protective or thin gutta-percha tissue. Finally place wet sublimated gauze, ten to twenty thicknesses, over and round the surface, followed by ordinary bandaging. In the subsequent dressings a bath of the saline solution or a weak solution of mercuric bichloride is used, followed by a spray of dioxide of hydrogen, which aids in removing all pus and loose dead tissues. The surface is again dusted with the powder, and protective strips, gauze and bandages applied. The use of formaldehyde for disinfecting septic wounds has, in the hands of G. E. Brewer,[4] in a large number of cases been followed with satisfactory results. But with regard to aseptic wounds, with a view of preventing infection, its attempted employment in but a single instance resulted in delayed union. The same disinfectant has been the subject of considerable experiments by C. Harrington[5] for the sterilisation of catgut, without diminishing its flexibility and tensile strength. Sterilisation alone is easily accomplished, but the methods successfully employed hitherto, have, as a rule, been found to so seriously impair these properties as to render the gut worthless for surgical purposes. Thoroughly infected gut was placed in a glass cabinet of 200,000 c.c. capacity, and subjected to a vapour of 5 c.c. formalin for a time varying from four to twenty-four hours. At the end of the exposure the gut proved to be sterile in all cases, while the results of the strength tests indicated, so far as could be judged in the absence of a fixed normal standard of strength for each size of catgut used, that neither tensile strength nor flexibility had been at all impaired. Professor Friedrich, of Leipsic, has recently[6] shown by experiments on animals that the infection of fresh wounds remains superficial for from six to eight hours. He concludes, therefore, that the proper treatment of open wounds not yet six hours old was to freshen the cut surfaces and stitch the edges accurately together. When the wound is more than eight hours old it should be packed and treated without stitching. Dr. Noetzel has confirmed Schimmelbusch's conclusions, that from fresh wounds bacteria were absorbed with wonderful rapidity. Ten minutes after infecting a wound with anthrax bacilli he had discovered them in the body. But he believes, nevertheless, that those which are so rapidly taken into the circulation always died, and that probably this quick absorption was a measure of self-protection on the part of the body. In speaking at the recent German Surgical Congress of the marvellous improvements of the aseptic in substitution of the antiseptic methods in surgery, Professor Mikulicz[7] again insisted upon the wearing of sterilised white thread gloves by the surgeon and his assistants to avoid infection of operation wounds. To protect the wounds from germs coming from the mouth he and his assistants cover their mouths with gauze bandages. He stated that this precaution was necessary because in 33 per cent. of 48 cases bacteriological examination showed that staphylococcus aureus was present in the mouths of healthy persons. Speaking, therefore, during the course of an operation ought to be avoided as much as possible. Dr. E. M. Foote[8] thinks that silk operating gloves are decidedly superior to rubber ones, and much less clumsy to use. In order to avoid infection of the wound from the hands he observes the following points: Keep the hands as far as possible out of the wound—wash and dry them and cover with sterilised gloves—for operations on the serous cavities and for difficult dissections; sterilise the hands by the well-known methods before covering them with the sterilised gloves. Quénu,[9] whilst admitting the difficulty of rendering the hands pure, attaches more importance to strict avoidance of contact with any septic patient for twenty-four hours before performing a serious operation. The ideal would be for the operating surgeon never to open an abscess, or, at least, never to touch pus.

[1] Berl. Klin. Woch, 1898, No. 10, and Les Nouv. Remèdes, May 24, 1898, p. 220. [2] Lancet, April [illegible]0, 1898. [3] Philadelphia Policlinic and Internat. Med. Mag., Mar., 1898, p. 224. [4] New York Med. Rec., Mar. 26, 1893, p. 435. [5] Amer. Jour. Med. Sci., May, 1898, d.544. [6] New York Med. Rec., May 7, 1898, p. 677. [7] Lancet, May 7, 1898, p. 1,294. [8] New York Med. Rec., April 16, 1898, p. [illegible]65. [9] Rev. de Chir., Mar., 1898, and Epit. Brit. Med. Jour., April 16, 1898.

